# Hexane-Isopropanolic Extract of Tungrymbai, a North-East Indian fermented soybean food prevents hepatic steatosis via regulating AMPK-mediated SREBP/FAS/ACC/HMGCR and PPARα/CPT1A/UCP2 pathways

**DOI:** 10.1038/s41598-018-27607-7

**Published:** 2018-07-03

**Authors:** Anjum Dihingia, Jijnasa Bordoloi, Prachurjya Dutta, Jatin Kalita, Prasenjit Manna

**Affiliations:** 1 Biological Science and Technology Division, CSIR-North East Institute of Science and Technology, Jorhat, Assam India; 2 Academy of Scientific and Innovative Research (AcSIR), CSIR-NEIST Campus, Jorhat, Assam India

## Abstract

This study for the first time examined the prophylactic role of Tungrymbai, a well-known fermented soybean food of North-East India, against hepatic steatosis. Treatment with hexane-isopropanolic (2:1, HIET) but not hydro-alcoholic (70% ethanol, HAET) extract dose-dependently (0.1, 0.2, or 0.3 µg/mL) reduced the intracellular lipid accumulation as shown by lower triglyceride levels and both Oil Red O and Nile Red staining in palmitate (PA, 0.75 mM)-treated hepatocytes. Immunobloting, mRNA expression, and knock-down studies demonstrated the role of AMPK-mediated SREBP/FAS/ACC/HMGCR and PPARα/CPT1A/UCP2 signaling pathways in facilitating the beneficial role of HIET against lipid accumulation in PA-treated hepatocytes. Animal studies further showed a positive effect of HIET (20 µg/kg BW, 8 weeks, daily) in regulating AMPK/SREBP/PPARα signaling pathways and reducing body weight gain, plasma lipid levels, and hepatic steatosis in high fat diet (HFD)-fed mice. Histological analyses also revealed the beneficial effect of HIET in reducing hepatic fat accumulation in HFD mice. Chemical profiling (HRMS, IR, and HPLC) demonstrated the presence of menaquinone-7 (vitamin K2) as one of the bio-active principle(s) in HIET. Combining all, this study demonstrates the positive effect of HIET on reducing hepatic steatosis via regulating AMPK/SREBP/PPARα signaling pathway.

## Introduction

The liver plays a major role in modulating lipid metabolism and maintaining lipid homeostasis. Non-alcoholic fatty liver disease (NAFLD) is gaining acceptance as a major health burden in the developed countries. It is highly associated with obesity and insulin resistance and is considered as the hepatic manifestation of metabolic syndrome^1–3^. NAFLD encompasses a broad spectrum of liver abnormalities ranging from simple hepatic steatosis (intrahepatic accumulation of triglyceride) to a more severe form, steatohepatitis, which is characterized by hepatocyte damage, chronic inflammation, and fibrosis and may progress to cirrhosis and liver failure^3^. The mechanism underlying the development of NAFLD includes an increased flow of free fatty acids, less elimination of triglyceride, and decreased fatty acid oxidation leading to an increase in *de novo* lipogenesis, suggesting that altered lipid metabolism contributes to the development of this disease^4^. Therefore, preventing hepatic fat accumulation and/or *de novo* lipogenesis and increasing the level of fatty acid oxidation may be helpful in improving the pathogenesis of NAFLD. To date, no standard treatment has been recognized for the effective management of NAFLD except lifestyle modification, increased physical activities, and improvement in diet quality.

Fermented soybean foods have long been consumed as complements for grain proteins in East and South-East Asian countries^5,6^. Various studies reported that fermented soybean foods and their functional components, including isoflavonoids, unsaturated fatty acids, and small peptides, could provide protection against chronic diseases, like obesity, type 2 diabetes, hypertension and carcinogenesis^5,6^. Fermenting the soybean has been found to enhance its nutritional value and efficacy^6^. North-East India is enriched with a variety of fermented soybean foods^7^. Tungrymbai is a well-known naturally fermented soybean food commonly consumed in the state of Meghalaya, North- East India, and it serves as a cheap source of high protein food in local diet^8^. Mishra *et al*. reported that Lactobacillus sp. are predominant in the microflora of Tungrymbai and 16S rDNA sequence analyses revealed the strains as *Lactobacillus brevis* (K38A and RD10) and *Lactobacillus fermentum* (K20)^9^. Thokchom and Joshi (2012) reported the antibiotic resistance and probiotic properties of this fermented soybean^10^. So far there is no study in the literature investigating the prophylactic potential of Tungrymbai against the metabolic diseases including NAFLD. This study for the first time examined the beneficial role of Tungrymbai against hepatic steatosis using both fatty acid-treated hepatocytes and high fat diet-fed animal models. The molecular mechanism of lipid metabolism has been dissected by immunoblotting studies, mRNA expression analyses, and signal silencing approach. Furthermore, the bioactive principle has also been identified by using different spectroscopic and chromatographic analyses, like high resolution mass spectroscopy (HRMS), infrared spectroscopy (IR), and high-performance liquid chromatography (HPLC).

## Results

### Effects of the different extracts of Tungrymbai (HAET and HIET) on intracellular lipid accumulation in PA-treated hepatocytes

**Figure 1:**
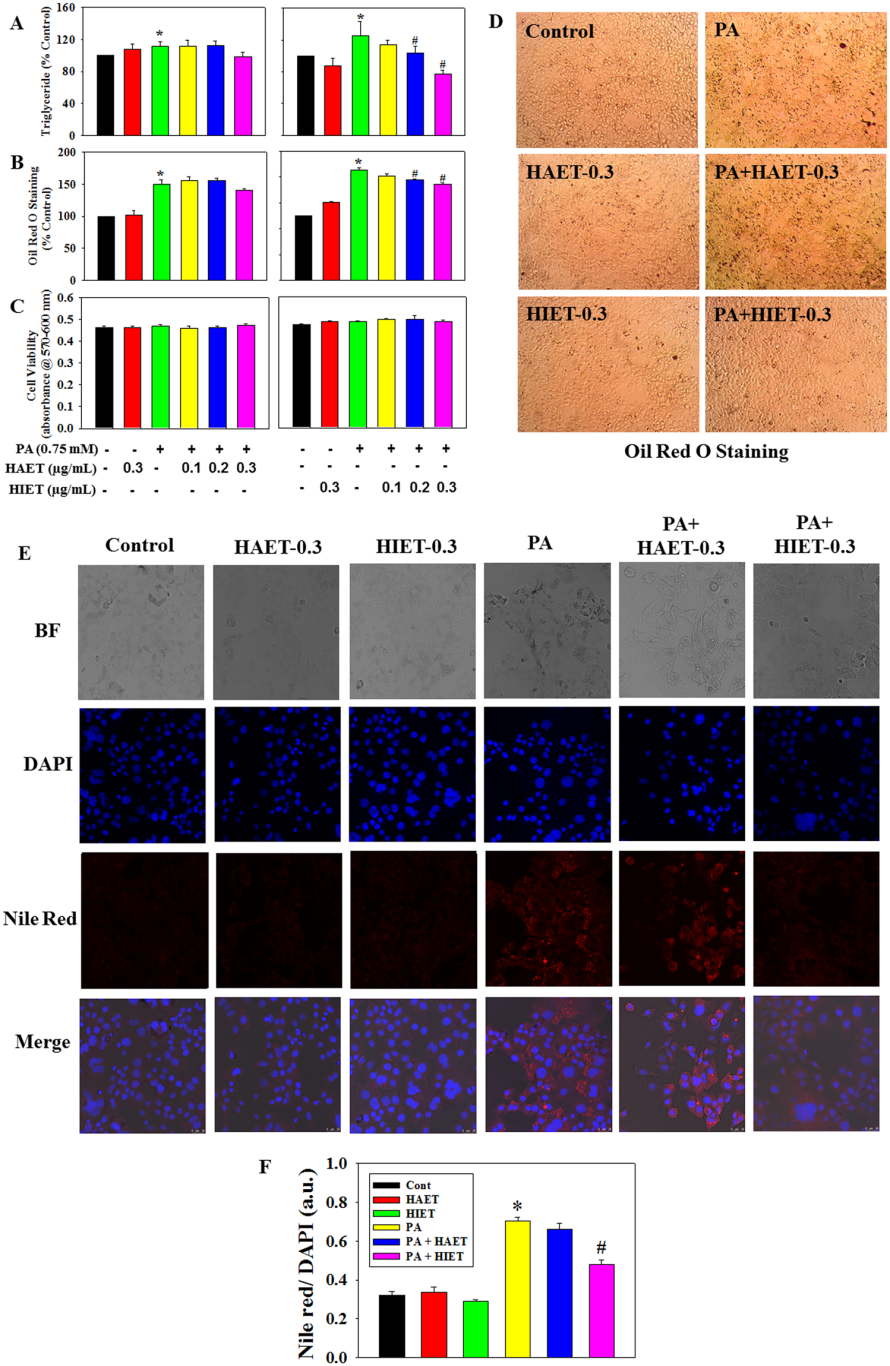
Effect of the different extracts of Tungrymbai, HAET (hydro-alcoholic, 70% ethanol) and HIET (hexane-isopropanol, 2:1) on intracellular lipid accumulation in palmitate (PA)-treated hepatocytes. (**A**) triglyceride content; (**B**) levels of Oil Red O staining; (**C**) cell viability; (**D**) representative photomicrographs of Oil Red O staining (10×); (**E**) representative photomicrographs of Nile Red and DAPI staining (40×); and (**F**) quantification of Nile Red/DAPI staining. Cells were pre-treated with HAET or HIET (0.1, 0.2, or 0.3 µg/mL) for 2 h followed by PA (0.75 mM) exposure for the next 20 h. Data are expressed as mean ± SE (n = 6). “*” Denotes the significant difference from untreated (*p < 0.05) and “#” denotes the significant difference from PA-treated groups (^**#**^*p* < 0.05).

To determine the effect of hydro-alcoholic (70% ethanol, HAET) and hexane-isopropanolic (2:1, HIET) extracts of Tungrymbai on intracellular lipid accumulation, hepatocytes were treated with different doses of HAET and HIET (0.1, 0.2, or 0.3 µg/mL) with or without palmitate (PA, 0.75 mM). Results showed that PA exposure significantly up-regulated the cellular triglyceride level compared to those seen in control group. Supplementation with HIET dose-dependently decreased the triglyceride level in PA-treated cells; however, no such significant decreases were observed in HAET-treated cells against PA exposure (Fig. 1A). Intracellular lipid accumulation was further assessed by Oil Red O staining, a well-known dye to stain cellular lipid droplets. Results showed that treatment with HIET but not HAET significantly inhibited the Oil Red O accumulation in PA-treated hepatocytes in a dose-dependent manner (Fig. 1B,D). Different treatments did not cause any change in cell viability level as examined by alamar blue reduction bioassay (Fig. 1C). Intracellular lipid vacuoles visible under phase-contrast microscopy were further confirmed by Nile Red/DAPI staining (Fig. 1E,F). Supplementation with HIET at a dose of 0.3 µg/mL significantly reduced the Nile Red accumulation in PA-treated cells; however, treatment with HAET did not show any significant effect compared to those seen in PA alone treated group. None of the treatment caused any changes in DAPI staining, which suggests unaltered cell viability throughout the study and this is in agreement with alamar blue study. Neither HIET nor HAET caused any changes in lipid accumulation in control cells. These studies suggest that treatment with HIET plays a beneficial role in preventing intracellular lipid accumulation in PA-treated cells.

### Effect of HIET on the signaling cascade of lipid metabolism in PA-treated hepatocytes

**Figure 2:**
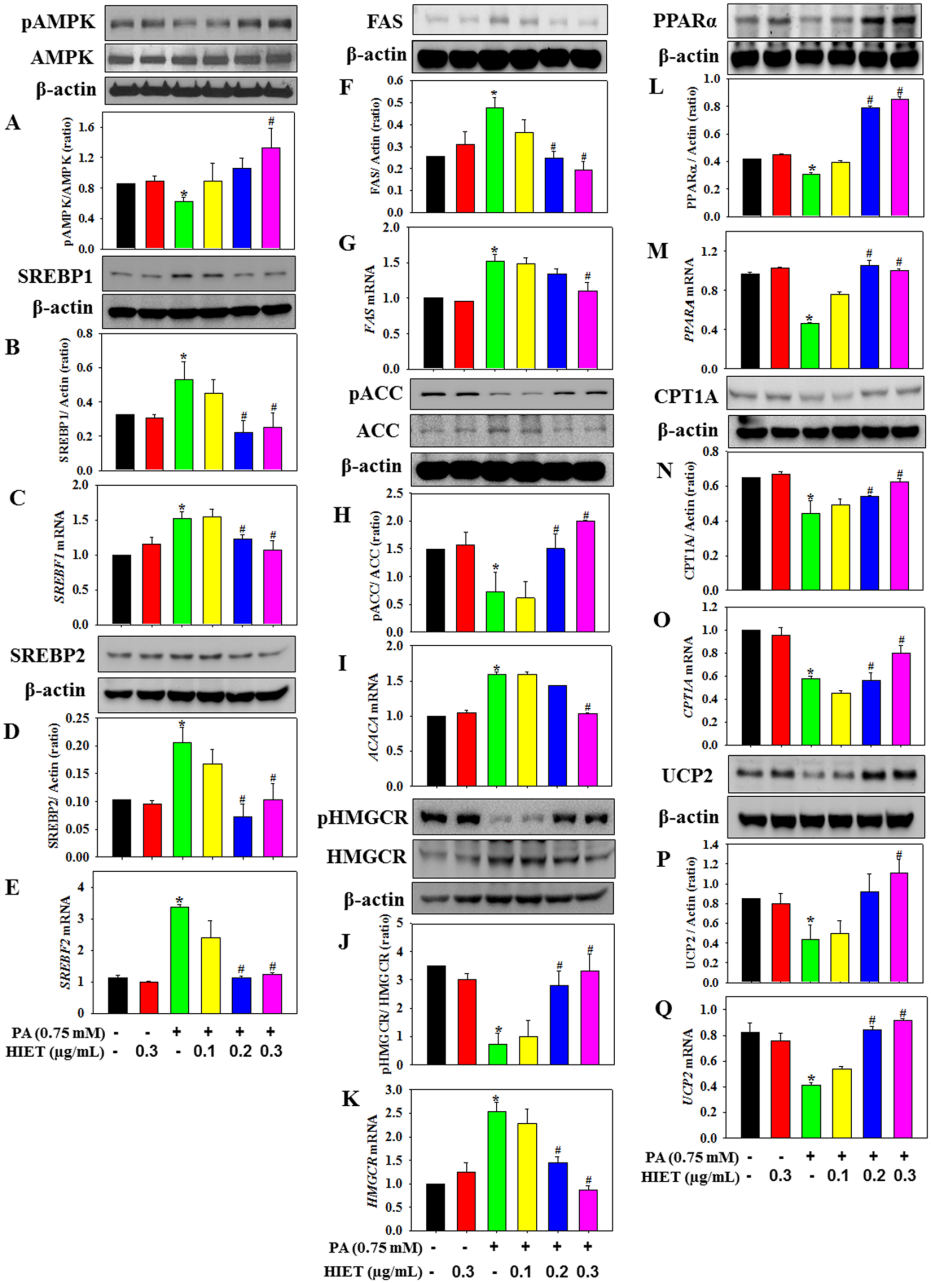
Effect of the hexane-isopropanolic extract (HIET) of Tungrymbai on the protein and mRNA expressions of different signaling molecules involved in lipid metabolism in palmitate (PA)-treated hepatocytes. (**A**) Phospho AMPK/AMPK protein expression; (**B**) SREBP1 protein expression; (**C**) *SREBF1* mRNA; (**D)** SREBP2 protein expression; (**E**) *SREBF2* mRNA; (**F**) FAS protein expression; (**G**) *FAS* mRNA; (**H**) phospho ACC/ACC protein expression; (**I**) *ACACA* mRNA; (**J**) phospho HMGCR/HMGCR protein expression; (**K**) *HMGCR* mRNA; (**L**) PPARα protein expression; (**M**) *PPARA* mRNA; (**N**) CPT1A protein expression; (**O**) *CPT1A* mRNA; (**P**) UCP2 protein expression; and (**Q**) *UCP2* mRNA. The blots are representatives of three independent experiments. Full-length blots are presented in Supplementary Figure 2. Cells were pre-treated with HIET (0.1, 0.2, or 0.3 µg/mL) for 2 h followed by PA (0.75 mM) exposure for the next 20 h. Data are expressed as mean ± SE. “*” Denotes the significant difference from untreated (*p < 0.05) and “#” denotes the significant difference from PA-treated groups (^**#**^*p* < 0.05).

Both immunoblotting and quantitative PCR studies have been carried out using hepatocyte cell culture model to dissect the molecular mechanism underlying the lipid lowering efficacy of HIET in PA-treated cells. Figure 2 showed that in PA-treated cells treatment with HIET dose dependently (0.1, 0.2, or 0.3 µg/mL) increased the protein expression of phospho AMPK, a key regulatory molecule involved in the lipid metabolism pathway. Supplementation with HIET also caused a decrease in the protein expressions of SREBP1, FAS, and SREBP2 and an increase in phospho ACC/ACC and phospho HMGCR/HMGCR ratio involved in the *de novo* lipogenesis and cholesterol deposition in PA-treated cells. Furthermore, HIET treatment increased the protein expression of PPARα, CPT1A, and UCP2 involved in the fatty-acid oxidation pathway. We also investigated the effects of HIET on the mRNA levels of genes involved in the lipid metabolism pathway. Results showed that HIET supplementation dose-dependently down-regulated the mRNA expressions of *SREBF1*, *FAS*, *ACACA*, *SREBF2*, and *HMGCR* and up-regulated the mRNA expressions of *PPARA*, *CPT1A*, and *UCP2* in PA-treated cells.

**Figure 3:**
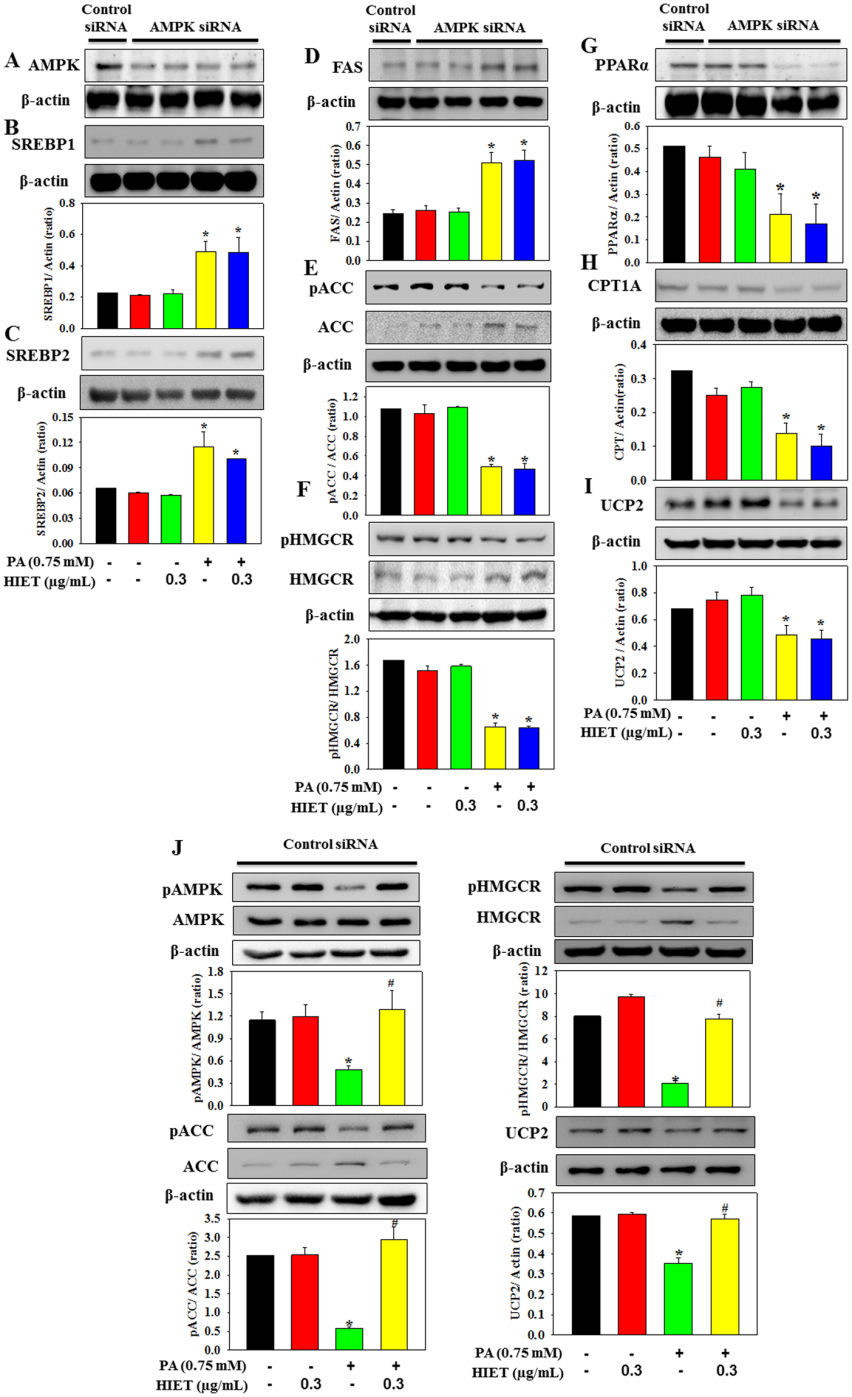
Effect of the hexane-isopropanolic extract of Tungrymbai, HIET on the protein expressions of different signaling molecules involved in lipid metabolism in palmitate (PA)-treated hepatocytes after transfection with either AMPK or control siRNA (100 nM). (**A**) AMPK; (**B**) SREBP1; (**C**) SREBP2; (**D**) FAS; (**E**) phospho ACC/ACC; (**F**) phospho HMGCR/HMGCR; (**G**) PPARα; (**H**) CPT1A; and (**I**) UCP2 protein expression in AMPK siRNA transfected cells. (**J**) Phospho AMPK/AMPK, phospho ACC/ACC, phospho HMGCR/HMGCR, and UCP2 protein expression in control siRNA transfected cells. The blots are representatives of three independent experiments. Full-length blots are presented in Supplementary Figure 3. Cells transfected with either AMPK or control siRNA were treated with HIET (0.3 µg/mL) for 2 h followed by PA (0.75 mM) exposure for the next 20 h. Control siRNA is a scrambled nonspecific RNA duplex that shares no sequence homology with any of the genes. Data are expressed as mean ± SE. “*” Denotes the significant difference from untreated (*p < 0.05).

AMPK is a key regulatory molecule that modulates hepatic lipid metabolism via regulating the transcription factors, SREBP1/2 and PPARα, all of which govern the expression of lipid metabolic enzymes^11,12^. In the present study we further investigated the role of AMPK in mediating the effect of HIET on hepatic lipid accumulation against PA exposure by using AMPK siRNA (Fig. 3). Results showed that in AMPK knockdown cells, HIET supplementation could not prevent the PA-induced activation of SREBP1, FAS, ACC, SREBP2, and HMGCR and the downregulation of phospho ACC, phospho HMGCR, PPARα, CPT1A, and UCP2. Interestingly, in control siRNA transfected cells, HIET supplementation upregulated the protein expression of phospho AMPK, phospho ACC, phospho HMGCR, and UCP and downregulated ACC and HMGCR in PA-treated cells (Fig. 3J). This indicated that AMPK phosphorylation facilitated the beneficial role of HIET in mediating the lipid metabolism pathway. These cell signaling studies and knockdown experiment demonstrated a positive effect of HIET on reducing the cellular lipid accumulation via regulating the AMPK-mediated SREBP1/FAS/ACC/SREBP2/HMGCR and PPARα/CPT1A/UCP2 signaling pathways in PA-treated hepatocytes.

### Effect of HIET supplementation on body weight gain, food intake, plasma lipid levels, and the signaling pathways of hepatic steatosis in HFD-fed mice

The levels of body weight gain, food intake, and plasma lipid levels in all the experimental animals have been shown in Table 1. Cell culture studies demonstrated that treatment with HIET at a dose of 0.3 µg/mL significantly reduced the intracellular lipid accumulation. Mice weighing on average 30 g have a total blood volume of approximately 2 ml; therefore, 0.3 µg/mL is equal to 20 µg/kg BW, the dose of HIET used for animal experiment. Body weight of all experimental mice was measured weekly to determine the dose of HIET supplementation. Results demonstrated that the level of body weight gain was significantly higher in HFD-fed mice compared to normal group. Supplementation with HIET (20 µg/kg BW) significantly reduced the gain in body weight in HFD-fed mice. Moreover, a significant increase in both triglyceride and total cholesterol has also been observed in HFD-fed mice, which could be attenuated by the HIET supplementation. There was no significant difference in food intake among the groups. The blood levels of liver and kidney function tests did not differ significantly.

**Table 1:** Body weight gain, food intake, liver (ALP and AST) and kidney (creatinine) function test, triglyceride (TG), and total cholesterol (TC) levels in the experimental animals.

Parameters	Normal	HIET	HFD	HFD + HIET
Body weight gain (%)	5.35 ± 1.20	5.11 ± 0.81	12.29 ± 0.82*	7.85 ± 0.86^#^
Food intake (g/mouse/day)	2.54 ± 0.03	2.52 ± 0.014	2.5 ± 0.01	2.52 ± 0.004
ALP (U/L)	38.88 ± 2.29	35.23 ± 1.64	38.93 ± 4.34	37.06 ± 3.83
AST (U/L)	27.81 ± 1.27	32.01 ± 6.26	34.92 ± 5.33	34.56 ± 2.11
Creatinine (mg/dL)	0.047 ± 0.0015	0.051 ± 0.0039	0.049 ± 0.0047	0.046 ± 0.0021
Triglyceride (mM)	0.57 ± 0.009	0.66 ± 0.03	1.9 ± 0.209*	1.18 ± 0.062^#^
Total Cholesterol (mM)	0.28 ± 0.019	0.32 ± 0.047	0.44 ± 0.059*	0.34 ± 0.055

Both normal and HFD-fed mice were gavaged with either olive oil or HIET (20 µg/kg BW, daily for 8 wks) respectively. Values are mean ± SE. “*” Denotes the significant difference from Normal (*p < 0.05) and “#” denotes the significant difference from HFD mice (^**#**^*p* < 0.05).

**Figure 4:**
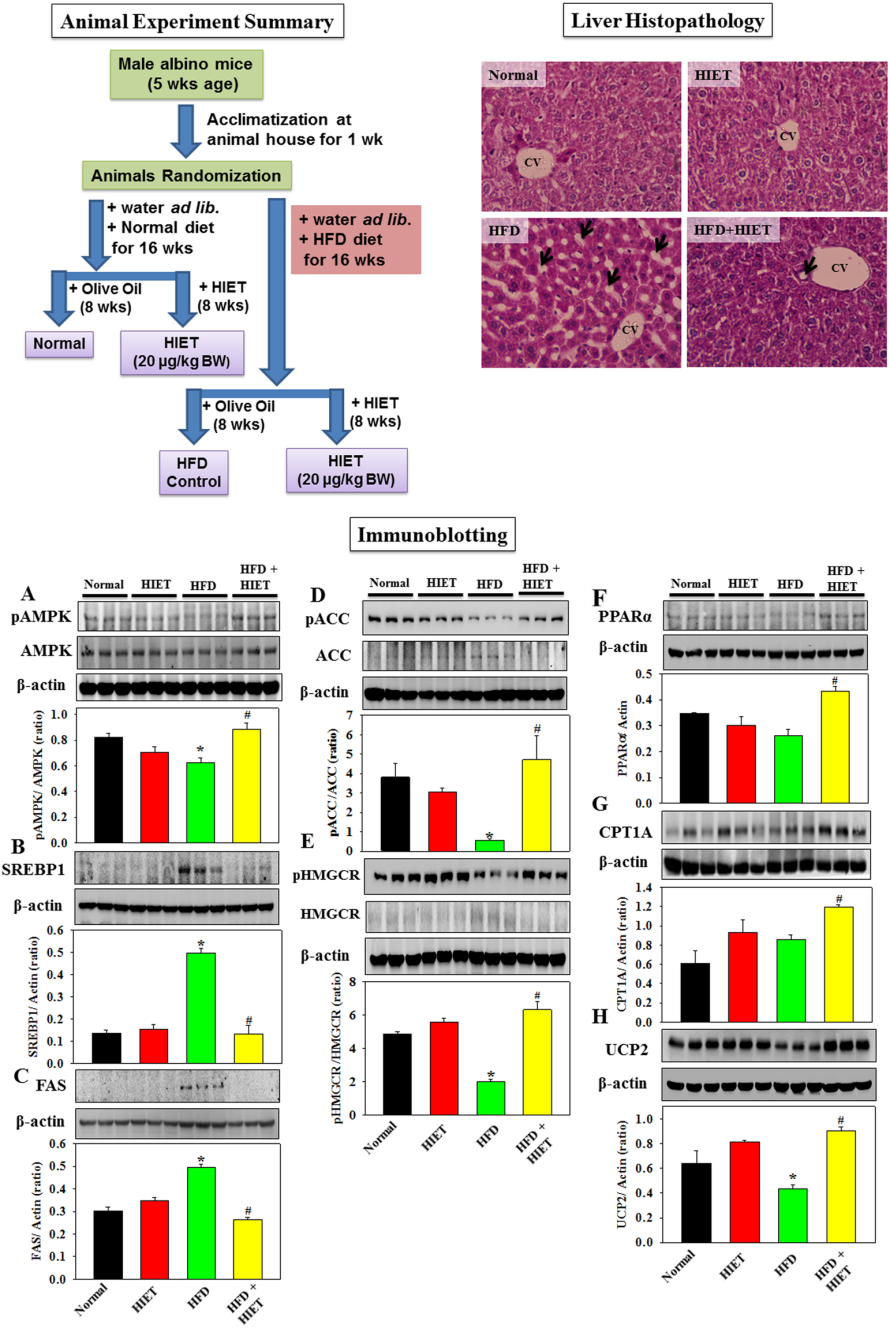
Schematic diagram of animal experiment and the effect of the hexane-isopropanolic extract of Tungrymbai, HIET on liver histology and the protein expressions of different signaling molecules in normal and high fat diet (HFD)-fed mice. Upper left panel represents the schematic diagram of animal experiment. Upper right panel represents the liver histology (40×) (arrows indicate lipid droplets). Lower panel represents the immunoblotting studies showing the protein expression of different signaling molecules including phospho AMPK/AMPK (**A**) SREBP1 (**B**) FAS (**C**) phospho ACC/ACC (**D**) phospho HMGCR/HMGCR (**E**) PPARα (**F**) CPT1A (**G**) and UCP2 (**H**). The blots are representatives of three independent experiments. Full-length blots are presented in Supplementary Figure 4. Normal mice were gavaged with HIET at a dose of 20 µg/kg BW (HIET) and HFD-mice were gavaged with HIET at a dose of 20 µg/kg BW (HFD + HIET) daily for 8 wks, each group consisting of five mice. Data are expressed as mean ± SE. “*” Denotes the significant difference from Normal (*p < 0.05) and “#” denotes the significant difference from HFD (^**#**^*p* < 0.05).

Figure 4 represents the effect of HIET on the protein expressions of different signaling molecules involved in lipid metabolism in the liver tissues of normal diet and HFD-fed mice. Results demonstrated that HIET supplementation up-regulated the AMPK phosphorylation and caused a decrease in the protein expression of SREBP1, FAS, ACC, and HMGCR and an increase in the protein expression of phospho ACC, phospho HMGCR, PPARα, CPT1A, and UCP2 in the liver tissues of HFD-fed mice. Treatment with HIET did not cause any changes in the protein expressions of all the above mentioned signaling molecules in normal diet-fed mice. Histological assessment further showed the appearance of fat infiltration in the liver tissue of HFD-fed mice compared to those seen in normal diet-fed mice. Supplementation with HIET significantly reduced the hepatic steatosis in the HFD-fed group.

### Spectroscopic and chromatographic analyses of HIET

**Figure 5:**
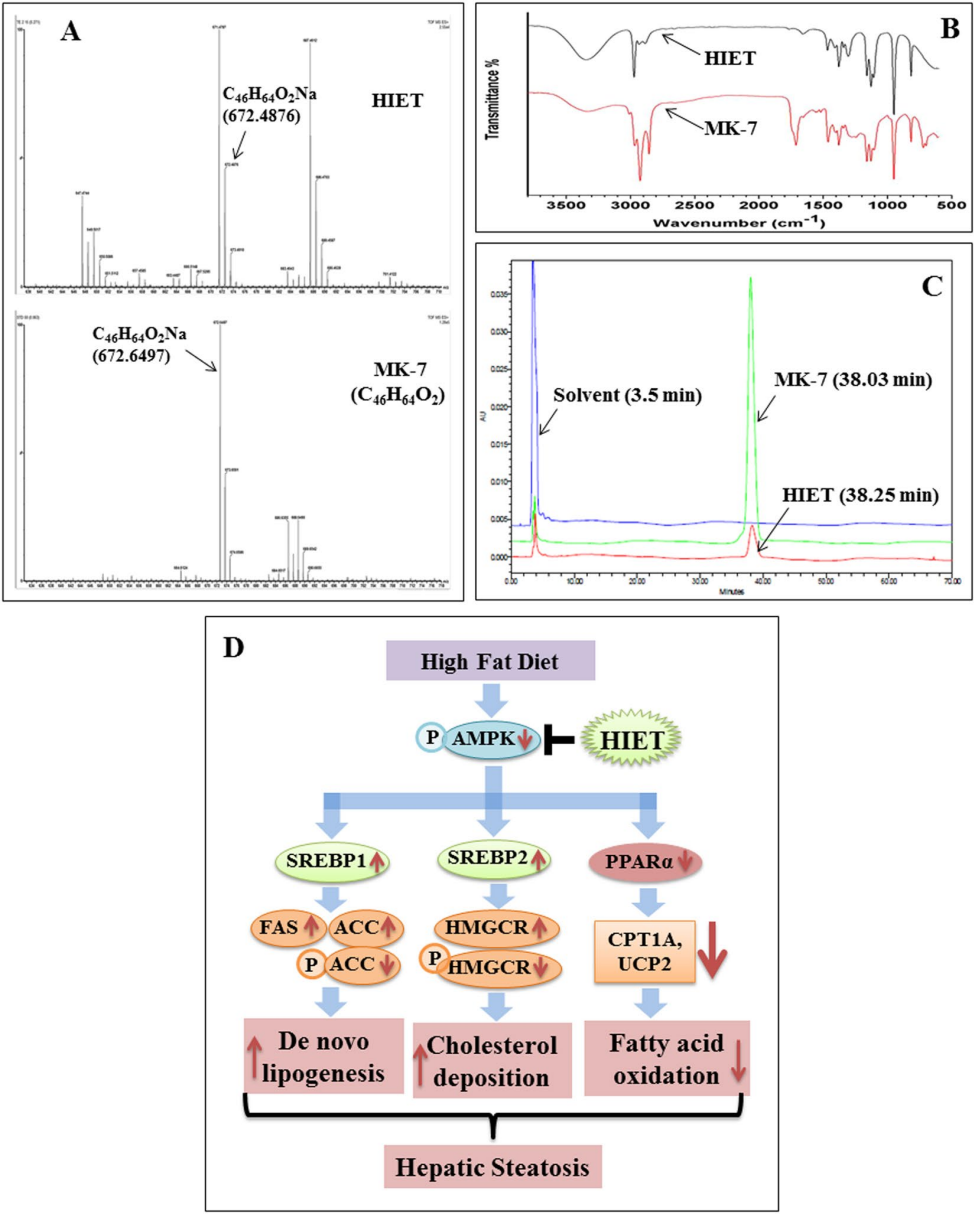
Chemical profiling of HIET and schematic diagram of proposed mechanism underlying the beneficial role of HIET against hepatic steatosis. (**A)** HRMS; (**B**) IR; (**C**) HPLC; and (**D**) proposed mechanism.

The spectroscopic (HRMS and IR) and chromatographic (HPLC) analyses of HIET have been represented in Fig. 5. HRMS analyses of HIET showed a peak at m/z 672.4876. HRMS analyses of standard menaquinone-7 (vitamin K2, C_46_H_64_O_2_, molecular weight 649.016) also showed a peak at m/z 672.6497 corresponding to C_46_H_64_O_2_Na, which suggest that menaquinone-7 may be one of the bio-active principle(s) present in HIET. The IR spectrum analysis of HIET demonstrated a fingerprint data with that of menaquinone-7 suggesting again the presence of menaquinone-7 in HIET. HPLC analyses of both HIET and menaquinone-7 showed a peak at a retention time of 38.25 and 38.03 min respectively, which further suggests the presence of menaquinone-7 as one of the bio-active component present in HIET. A peak around the retention time of 3.5 min represented the solvent (hexane-isopropanol mix) used for dissolving HIET and menaquinone-7.

## Discussion

Various studies reported a potentially significant role of East and South-East Asian fermented soybean foods in preventing metabolic diseases, including obesity, type 2 diabetes, and cardiovascular diseases^6^. North-East India is enriched with a variety of fermented soybean foods due to a diverse population of people with different ethnic background^7^. However, there is no report about the beneficial effect of the naturally fermented soybean foods of North-East India against metabolic diseases and its associated health disorders, like NAFLD. This study hypothesized that Tungrymbai, a well-known fermented soybean food commonly consumed in the state of Meghalaya, India, could prevent the intracellular lipid accumulation, impaired lipid oxidation, and hepatic steatosis via regulating AMPK/SREBP/PPARα signaling pathway.

In the present study, we first investigated the lipid lowering efficacy of the different extracts of Tungrymbai, namely HAET and HIET using palmitate (PA)-treated hepatocyte cell culture model. Results showed that treatment with HIET but not HAET dose-dependently (0.1, 0.2, or 0.3 µg/mL) reduced the intracellular triglyceride levels in PA-treated cells compared to those seen in PA-alone treated cells. Both Oil Red O and Nile red stains are well-known markers to detect the intracellular lipid accumulation^1,13^. Further studies with Oil Red O and Nile red staining also revealed that HIET but not HAET at a dose of 0.3 µg/mL significantly reduced the intracellular lipid content in PA-treated cells. Both HIET and HAET did not cause any changes in lipid accumulation in control cells. These studies suggest that treatment with HIET plays a beneficial role in preventing intracellular lipid accumulation in PA-treated cells.

AMPK is emerging as an important signaling molecule in the regulation of energy metabolism and metabolic syndrome associated chronic diseases^14^. Activation of AMPK modulates hepatic lipid metabolism via regulating several transcription factors, including SREBP1/2 and PPARα, all of which govern the expression of lipid metabolic enzymes^11,12^. The isoform, SREBP1 preferentially regulates the *de novo* lipogenesis by activating the genes, FAS and ACC, required for lipid synthesis whereas SREBP2 controls the cholesterol homeostasis via activating the gene, HMGCR, required for cholesterol synthesis^12^. PPARα, on the other hand, regulates the genes CPT1A and UCP2, which are involved in fatty acid oxidation^15^. Our study demonstrates that supplementation with HIET dose-dependently increased the AMPK phosphorylation, decreased the protein expressions of SREBP1, FAS, ACC, SREBP2, and HMGCR and up-regulated the protein expressions of phospho ACC, phospho HMGCR, PPARα, CPT1A, and UCP2 in PA-treated cells. Treatment with HIET also caused a decrease in the mRNA expressions of *SREBF1*, *FAS*, *ACACA*, *SREBF2*, and *HMGCR* and an increase in *PPARA*, *CPT1A*, and *UCP2* in PA-treated cells. Interestingly, in AMPK-knockdown cells HIET did not down-regulate the activation of SREBP1, FAS, ACC, SREBP2, and HMGCR and up-regulate the phospho ACC, phospho HMGCR, PPARα, CPT1A, and UCP2 protein expression in PA-treated cells. These cell culture studies suggest that activation of AMPK-mediated SREBP1/FAS/ACC/SREBP2/HMGCR and PPARα/CPT1A/UCP2 signaling pathways plays an important role in mediating the prophylactic role of HIET against PA-induced intracellular lipid accumulation.

In addition to the cell culture studies, we also examined the beneficial role of HIET against hepatic lipid accumulation by using HFD-fed animal model. Results demonstrated that supplementation with HIET (20 µg/kg BW, 8 weeks, daily) had a positive effect on lowering body weight gain and circulating triglyceride and cholesterol levels in HFD-fed mice compared to those seen in HFD-fed group. Immunobloting studies further showed that treatment with HIET significantly upregulated the AMPK phosphorylation, down-regulated the protein expressions of SREBP1, FAS, ACC, and HMGCR, and up-regulated phospho ACC, phospho HMGCR, PPARα, CPT1A, and UCP2 in the liver tissue of HFD-fed mice. Histological studies again revealed the hepatic steatosis in the HFD-fed group compared to normal diet-fed group, and fat infiltration was significantly reduced in the liver tissue collected from the HIET-treated HFD-fed mice. Our animal study also demonstrated a beneficial role of HIET in preventing the HFD-induced hepatic steatosis via regulating AMPK/SREBP/PPARα signaling pathway.

The present study further examined the bio-active principle(s) present in HIET by using different spectroscopic (HRMS, IR) and chromatographic (HPLC) analyses. Results showed the presence of menaquinone-7, commonly known as vitamin K2 as one of the bioactive principles present in HIET. Various animal studies reported the beneficial role of vitamin K2 in reducing plasma levels of total cholesterol and triglyceride^16–18^. Moreover, the lipid lowering effect of vitamin K2 has also been observed among patients with continuous ambulatory peritoneal dialysis^19^. The present study suggests that menaquinone-7 in HIET may play an important role in mediating the beneficial effect of HIET on lowering hepatic steatosis.

In conclusion, this is the first report to demonstrate that supplementation with a hexane-isopropanolic extract of Tungrymbai decreased the PA-induced intracellular lipid accumulation as manifested by lower triglyceride level and Oil Red O/Nile Red staining in hepatocytes. Immunoblotting studies, mRNA expression analyses, and signal silencing studies showed that AMPK/SREBP/PPARα signaling pathway plays an important role in mediating the prophylactic role of HIET against PA-induced intracellular lipid accumulation. Moreover, animal studies along with histological findings also demonstrated the beneficial role of HIET in reducing body weight gain, plasma lipid levels, and regulating the AMPK/SREBP/PPARα signaling cascade to reduce hepatic steatosis. Chemical profiling studies including HRMS, IR, and HPLC further depicted the presence of menaquinone-7 as one of the bio-active principles in HIET. Figure 5D demonstrates the proposed mechanism underlying the prophylactic role of HIET on lowering hepatic steatosis. The findings about the link between HIET, AMPK/SREBP/PPARα, and hepatic lipid metabolism may be helpful for the development of a novel adjuvant therapy to achieve better control of NAFLD and improve the lives of the patient population.

## Materials and Methods

### Materials

Anti-AMPK (#5831S), anti-phosphorylated AMPK (Thr 172) (#2535S), and anti-phosphorylated ACC (Ser 79) (#3661S) were purchased from Cell Signaling Technology (Beverly, MA, USA). Anti-ACC (#NB100–92011) primary antibody was purchased from Novus Biologicals (Littleton, CO, USA). Anti-CPT1A (#ab128568), anti-FAS (#ab22759), anti-HMGCR (#ab174830), anti-phosphorylated HMGCR (Ser 872) (#ab78275), anti-PPARα (#ab8934), anti-SREBP1 (#ab28481), and anti-SREBP2 (#ab30682) primary antibodies were purchased from Abcam, Inc. (Cambridge, MA, USA). Anti-UCP2 (#662047) primary antibody was purchased from Merck (Darmstadt, Germany). Pre-designed primers for *SREBF1* (#FR1_Srebf1), *SREBF2* (#FR1_Srebf2), *FAS* (FR1_Fas), *ACACA* (FR1_Acaca), *HMGCR* (FR1_Hmgcr), *PPARA* (FR1_Ppara), *CPT1A* (FR1_Cpt1a), *UCP2* (FR1_Ucp2), or *ACTIN-B* (FR1_Actb) were purchased from Sigma Chemical Co. (St. Louis, MO, USA). All chemicals were purchased from Sigma unless otherwise mentioned.

### Sample collection and extraction of Tungrymbai

The samples of fermented soybean, Tungrymbai were collected from the local market of Umsning, Ri Bhoi district, Meghalaya, India (Geographic coordinates: ~25°74′68.06′′ North and 91°88′91.94′′ East) in July 2016. Samples were collected aseptically in pre-sterile poly-bags, sealed, and stored at −80 °C for further analyses. Two extracts, namely hydro-alcoholic (70% ethanol, HAET) and hexane-isopropanolic (2:1, HIET) were prepared by using soxhlet apparatus. The samples were extracted three times using respective solvent in a ratio of 1:3 (w/v) at room temperature. The extract was made solvent free using vacuum rotary evaporator and kept in −80 °C until used.

### Cell culture studies

#### Cell Culture

The rodent hepatocyte cell line, CC1 was obtained from Sigma. These cells were maintained at 37 °C in DMEM medium containing 5.5 mM glucose, 10% (v/v) heat-inactivated FBS, 1% non-essential amino acid (NEAA), 100 U/mL penicillin, 100 µg/mL streptomycin, 12 mM sodium bicarbonate, 20 mM HEPES and 2 mM glutamine in a humidified atmosphere containing 5% (v/v) CO_2_.

### Preparation of BSA conjugated palmitate solution

Cells were treated with palmitate as a conjugate with fatty acid free-BSA. The palmitate-BSA solution was prepared following the protocol as mentioned earlier^1^. Briefly, sodium palmitate was dissolved in 50% (v/v) ethanol, diluted in DMEM containing 2% (w/v) BSA, and the solution was incubated at 37 °C for 2 h with stirring to prepare BSA-conjugated palmitate solution (6:1 molar ratio of palmiate:BSA, which is close to the ratio observed in human serum^20^).

### Treatment of hepatocytes with palmitate and Tungrymbai extract

Cells were treated with palmitate (PA, 0.75 mM) with or without different extracts of Tungrymbai (HAET or HIET). Various concentrations (0.1, 0.2, or 0.3 µg/mL) of HAET or HIET were supplemented for 2 h followed by PA exposure for the next 20 h^21^. Control cells were treated with DMEM containing 2% BSA with or without 0.3 µg/mL of HAET or HIET. On termination of incubations, cells were lysed in radioimmunoprecipitation assay (RIPA) buffer (50 mM Tris pH 8, 150 mM NaCl, 1% NP-40, 0.5% deoxycholic acid, 0.1% SDS) supplemented with protease and phosphatase inhibitors (1 mM PMSF, 5 µg/mL leupeptin, 2 µg/mL aprotinin, 1 mM EDTA, 10 mM NaF, and 1 mM NaVO_4_). Lysates were cleared by centrifugation and total protein concentrations were determined by BCA assay (Pierce/Thermo Scientific, Rockford, IL).

### Cell viability and triglyceride assay

Cell viability was determined using the alamar blue reduction bioassay (HIMEDIA, Mumbai, India). This method is based upon Alamar Blue dye reduction by live cells^22^. The cellular triglyceride level was measured following the procedure as described earlier including total lipid extraction by chloroform-methanol extraction procedure followed by detection using commercial available kits from Robonik Pvt. Ltd. (Mumbai, India)^23^.

### Oil Red O and Nile Red/DAPI staining

Intracellular lipid accumulation was examined by using both Oil Red O and Nile Red/DAPI staining following the protocol as mentioned earlier^1,13^. For Oil Red O staining, cells were fixed with 4% paraformaldehyde in PBS for 15 min and then stained with 0.2% Oil Red O dissolved in 60% isopropanol for 10 min at room temperature followed by washing with distilled water^1^. Cells were imaged using a microscope (Carl Zeiss Inc, Germany). Next, cells were incubated with 100% isopropanol solution for 10 min at room temperature to elute Oil Red O reagent. The eluted solution was removed, centrifuged for 1 min at 12000 rpm to remove any cell materials, and measured the absorbance at 540 nm with a microplate reader (BioTek, USA). The result was expressed as percentage over control. For Nile Red/DAPI staining, cells were stained with Nile Red (0.2 mg/mL) for 15 min at room temperature after fixation with paraformaldehyde, washed with PBS, and mounted with cover slip using the mounting media containing DAPI^13^. Images were acquired by confocal microscopy with an inverted laser scanning confocal microscope and the cellular fluorescence was quantified using image analysis software (Leica Microsystems, Germany).

### Immunobloting

Samples containing approximately the same amount of protein (~20–40 µg) were run on 10 or 12% SDS-PAGE and transferred to a nitrocellulose membrane. Membranes were blocked at room temperature for 2 h in blocking buffer containing 1% BSA to prevent non-specific binding and then incubated with anti-ACC (1:1000), anti-phosphorylated ACC (1:1000), anti-AMPK (1:1000), anti-phosphorylated AMPK (Thr 172) (1:1000 dilution), anti-CPT1A (1:1000), anti-FAS (1:1000), anti-HMGCR (1:1000), anti-phosphorylated HMGCR (1:1000), anti-PPARα (1:1000), anti-SREBP1 (1:1000), and anti-SREBP2 (1:1000), or anti-UCP2 (1:1000) primary antibody at 4 °C overnight. Beta-actin (1:30000) was used as a loading control. The membranes were washed in TBS-T (50 mmol/L Tris-HCl, pH 7.6, 150 mmol/L NaCl, 0.1% Tween 20) for 30 min and incubated with the appropriate HRP conjugated secondary antibody (1:5000 dilution) for 2 h at room temperature, and developed using the ECL substrate (BioRad). The intensity of each immunoblotting band was measured using the histogram tool of Adobe Photoshop CS5.

### Analysis of mRNA expression by quantitative PCR

Total RNA was extracted from cells using TRIzol Reagent (Life Technologies) following the manufacturer’s instructions. The quality and the quantity of the extracted RNA were determined on Epoch2 microplate reader using Take3 micro volume plate (BioTek, USA). First-strand complementary DNA (cDNA) synthesis was performed using a commercially available High Capacity RNA-To-cDNA kit (Life Technologies) in a final reaction volume of 20 µL. Quantification of mRNA was performed by using PowerUP SYBR Green master mix (Invitrogen) on PikoReal-96 Real-time PCR (Thermo Scientific). PCR conditions were 2 min at 50 °C, 2 min at 95 °C, then 40 cycles of 95 °C for 15 s, 60 °C for 15 s, and 72 °C for 60 s. Primers (10 µM) were used in a final reaction volume of 10 µL. Beta-actin was used as a housekeeping gene to normalize threshold cycle (CT) values. To exclude nonspecific amplification and/or the formation of primer dimers, control reactions were performed in the absence of target cDNA. All of the experiments were run in triplicate. The relative amounts of mRNAs were calculated using the relative quantification (ΔΔCT) method.

### Signal Silencing Studies

AMPK-siRNA and control siRNA were purchased from Sigma (St. Louis, MO) and Santa Cruz Biotechnology, Inc. (Santa Cruz, CA) respectively. Cells were transiently transfected with 100 nM siRNA complex using Lipofectamine 2000 transfection reagent (Invitrogen, Carlsbad, CA) following the method as described earlier^22^. After transfection cells were treated with HIET and PA following the method as described above.

### Animal studies

#### Animals

Male Swiss albino mice (5 weeks old, 25–30 g) were obtained from Bose Institute animal research facility. All animals were kept in the animal care facility maintaining ambient environmental conditions (12:12-h light-dark cycle, 22–24 °C). Experiments were carried out according to the guidelines of the Institutional Animal Ethical Committee (IAEC), Bose Institute, Kolkata. The study was also approved by both CPCSEA (Committee for the Purpose of Control & Supervision on Experiments on Animals), Ministry of Environment & Forests, New Delhi, India (1796/PO/Ere/S/14/CPCSEA) and IAEC.

#### Animal experimental design

Mice were divided into four groups by computer-generated randomization, and each group contained five animals. The mice were fasted overnight before being weighed. HIET was dissolved in 0.1% olive oil (OO), and an aliquot of 0.1 mL of the stock solution was given per 100 g BW. The mice were treated as follows (Fig. 4A):

Normal - animals were fed a low fat diet (providing 10% calories as fat) for 16 weeks, and OO was administered at a dose of 100 µL/100 g BW daily by oral gavage for last 8 weeks.

HIET - animals were fed a low fat diet for 16 weeks, and HIET was administered at a dose of 20 µg/kg BW daily by oral gavage for last 8 weeks.

HFD - animals were fed a high fat diet (providing 45% calories as fat) for 16 weeks, and OO was administered at a dose of 100 µL/100 g BW daily by oral gavage for last 8 weeks.

HFD + HIET - animals were fed HFD for 16 weeks, and HIET was administered at a dose of 20 µg/kg BW daily by oral gavage for last 8 weeks.

The composition of the experimental diet has been provided in our earlier studies^24,25^. Body weight was measured weekly, and food intake was recorded daily. At the end of 16 weeks the animals were fasted overnight, weighed, and euthanized by exposure to isoflurane. Blood was collected via heart puncture with a 19^1/2^ gauge needle into EDTA Vacutainer tubes. Plasma was isolated after centrifuging the blood in a 4 °C centrifuge at 3000 rpm for 15 min. Liver tissues excised from the experimental animals were perfused with cold saline to remove leftover blood, weighed, and immediately stored at −80 °C until further use.

#### Measurement of plasma lipid profile and liver and kidney function test

Plasma lipid profile (triglyceride and total cholesterol) and liver (alkaline phosphatase and aspartate aminotransferase) and kidney (creatinine) function tests were examined by using kits from Robonik Pvt. Ltd. (Mumbai, India). All appropriate controls and standards as specified by each manufacturer’s kit were used.

### Histological Studies

Liver samples from the experimental mice were fixed in 10% buffered formalin and processed for paraffin sectioning. Sections of about 5 μm thickness were stained with hematoxylin and eosin, and images were acquired by microscope (Carl Zeiss Inc, Germany).

### Chemical profiling of HIET

The high resolution mass spectroscopy (HRMS), infrared spectroscopy (IR), and high-performance liquid chromatography (HPLC) analyses were performed to examine the chemical profiling of HIET. The HRMS analysis was performed on an ESI-Q-TOF micro mass spectrometer (Waters, Germany) in the electrospray ionization mode (positive mode). The IR spectrum analysis was done by placing a small amount of sample on the ATR accessory using an ALPHA FTIR spectrometer (Bruker, Germany). HPLC analysis was performed on a Waters^TM^ HPLC System (Waters, Germany) equipped with a C30 column (4.6 × 250 mm with 5 µm pore size) followed by Waters 2489 UV/Visible Detector (λ 248 nm). The mobile phase was composed of methanol:ethanol (95:5 v/v), with an elution volume of 0.5 ml/min. The temperature was maintained at 23 °C.

### Statistical analysis

Data were analyzed statistically using ANOVA with Sigma Stat statistical software (San Jose, CA, USA). When data passed a normality test, all groups were compared using the Student–Newman–Keuls method. A *p* value of less than 0.05 was considered significant for a statistical test.

## Electronic supplementary material

Supplementary Dataset 1
