# Supplementary material for: Hexane-Isopropanolic Extract of Tungrymbai, a North-East Indian fermented soybean food prevents hepatic steatosis via regulating AMPK-mediated SREBP/FAS/ACC/HMGCR and PPARα/CPT1A/UCP2 pathways
Source: Sci Rep. 2018 Jul 3;8:10021. doi: 10.1038/s41598-018-27607-7 (PMC6030226; doi:10.1038/s41598-018-27607-7)

**Hexane-Isopropanolic Extract of Tungrymbai, a North-East Indian fermented soybean food prevents hepatic steatosis via regulating AMPK-mediated SREBP/FAS/ACC/HMGCR and PPARα/CPT1A/UCP2 pathways**

**Anjum Dihingia^1,2,3^, Jijnasa Bordoloi^1,2,3^, Prachurjya Dutta^1,2,3^, Jatin Kalita^1,2^, Prasenjit Manna^1,2 *^**

^1^Biological Science and Technology Division, CSIR-North East Institute of Science and Technology, Jorhat, Assam, India

^2^Academy of Scientific and Innovative Research (AcSIR), CSIR-NEIST Campus, Jorhat, Assam, India

^3^Authors contributed equally in the study

**Running title:** *Prophylactic role of Tungrymbai against hepatic steatosis*

Address for Correspondence:

Dr. Prasenjit Manna, Biological Science and Technology Division, CSIR-North East Institute of Science and Technology, Jorhat, Assam, 785006, India; TEL: 91-376-2370012, FAX: 91-376-2370011, E-MAIL: pmanna2012@gmail.com

**Supplementary information: Full-length blots of immunoblotting data**


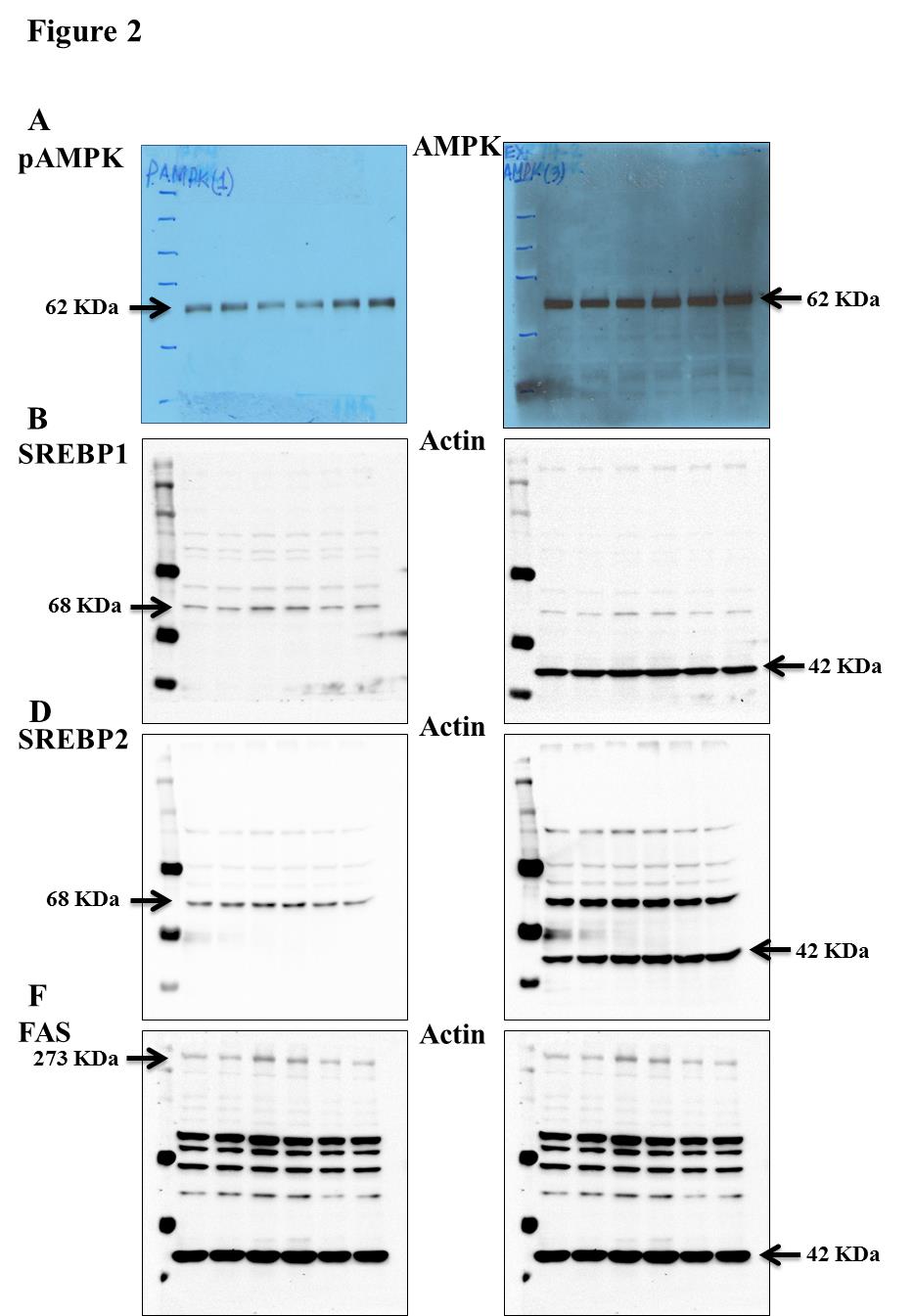


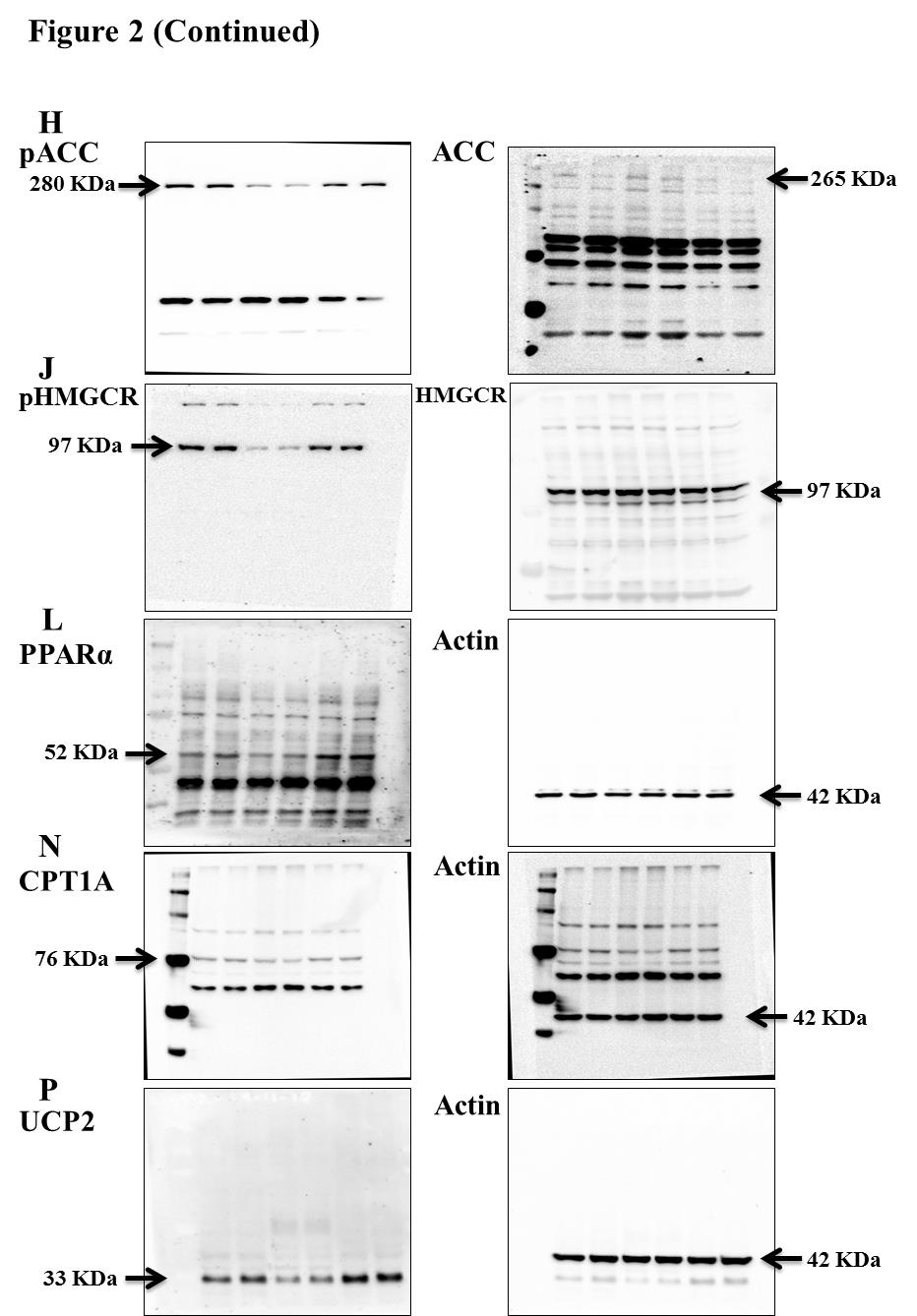


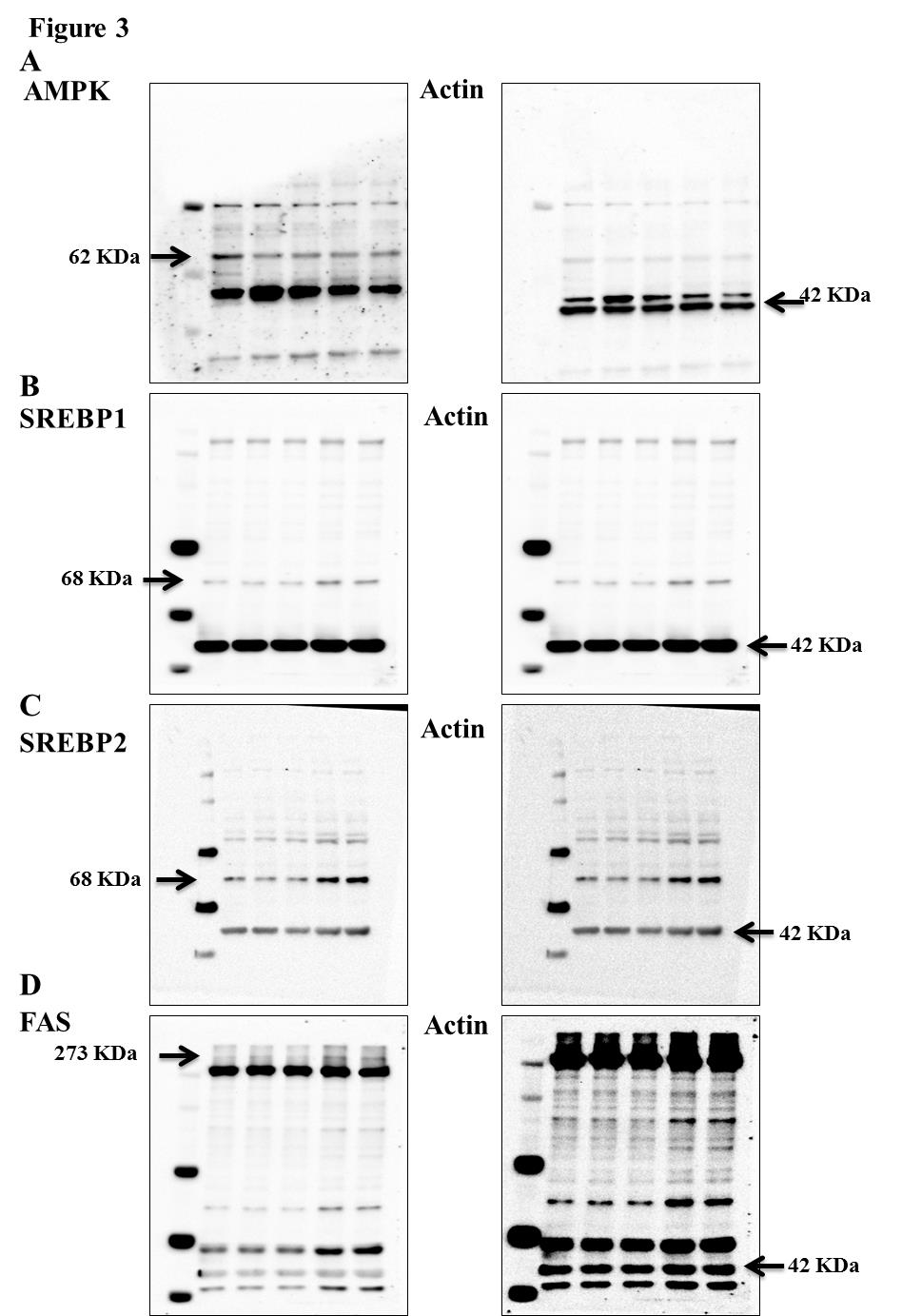


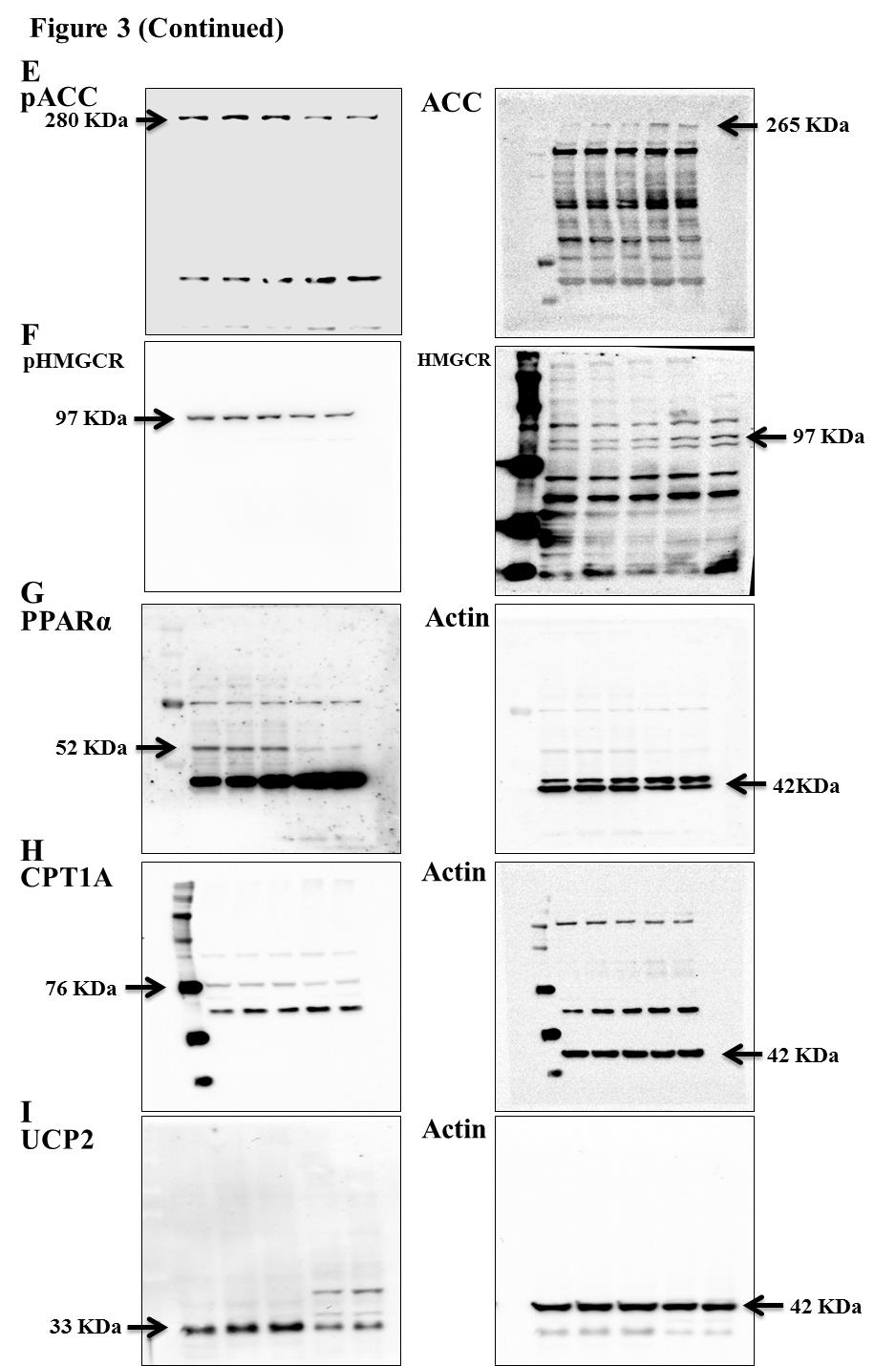


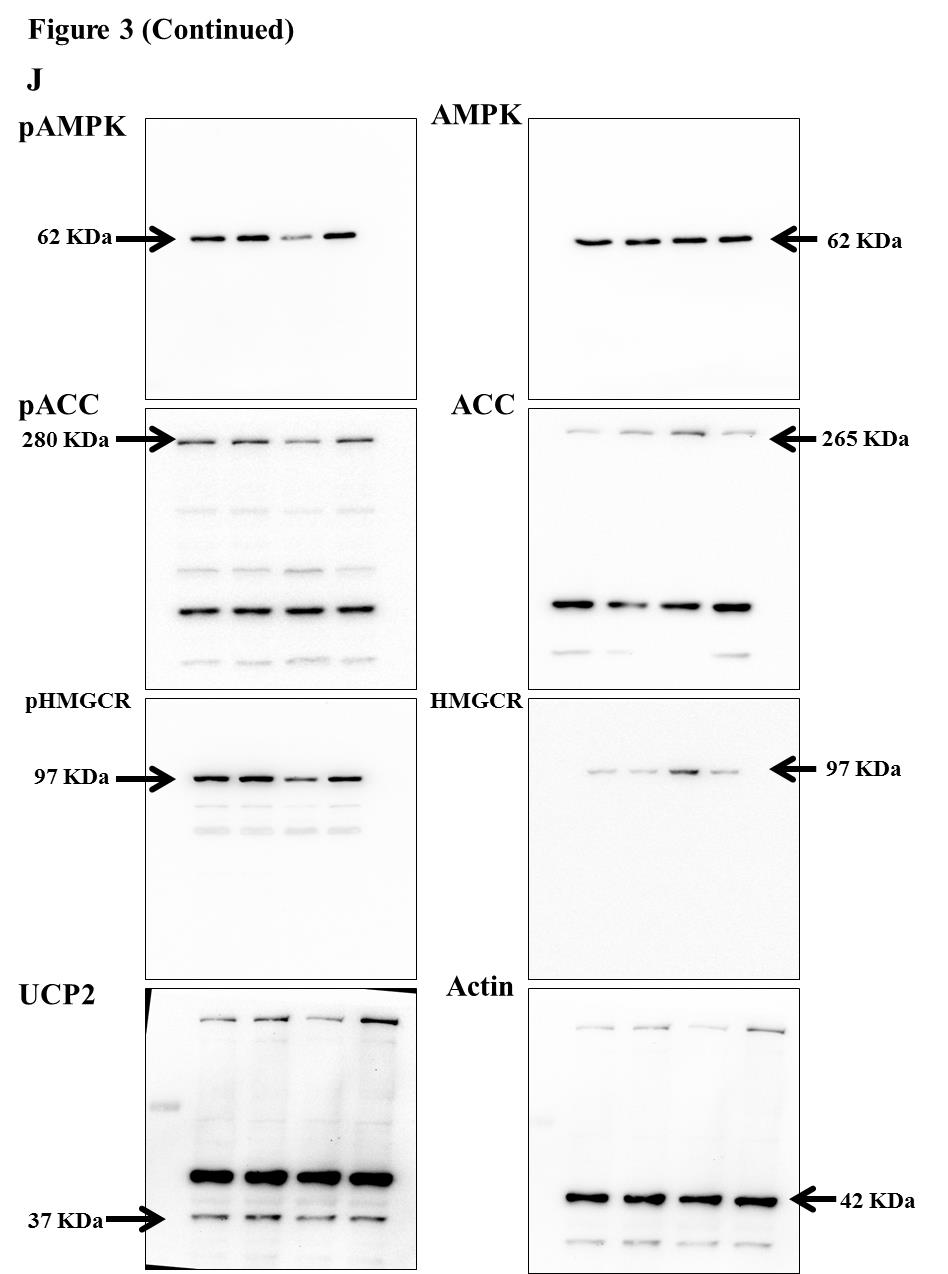


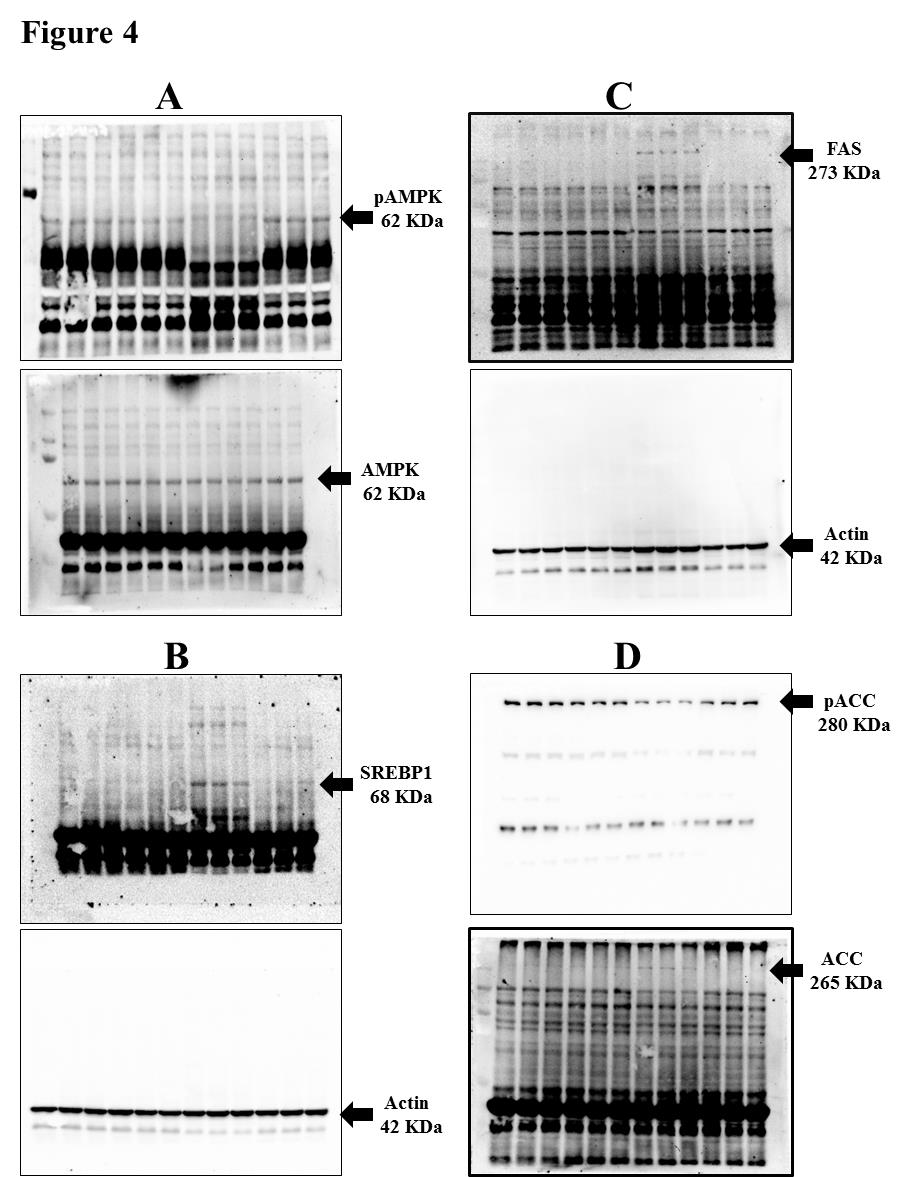


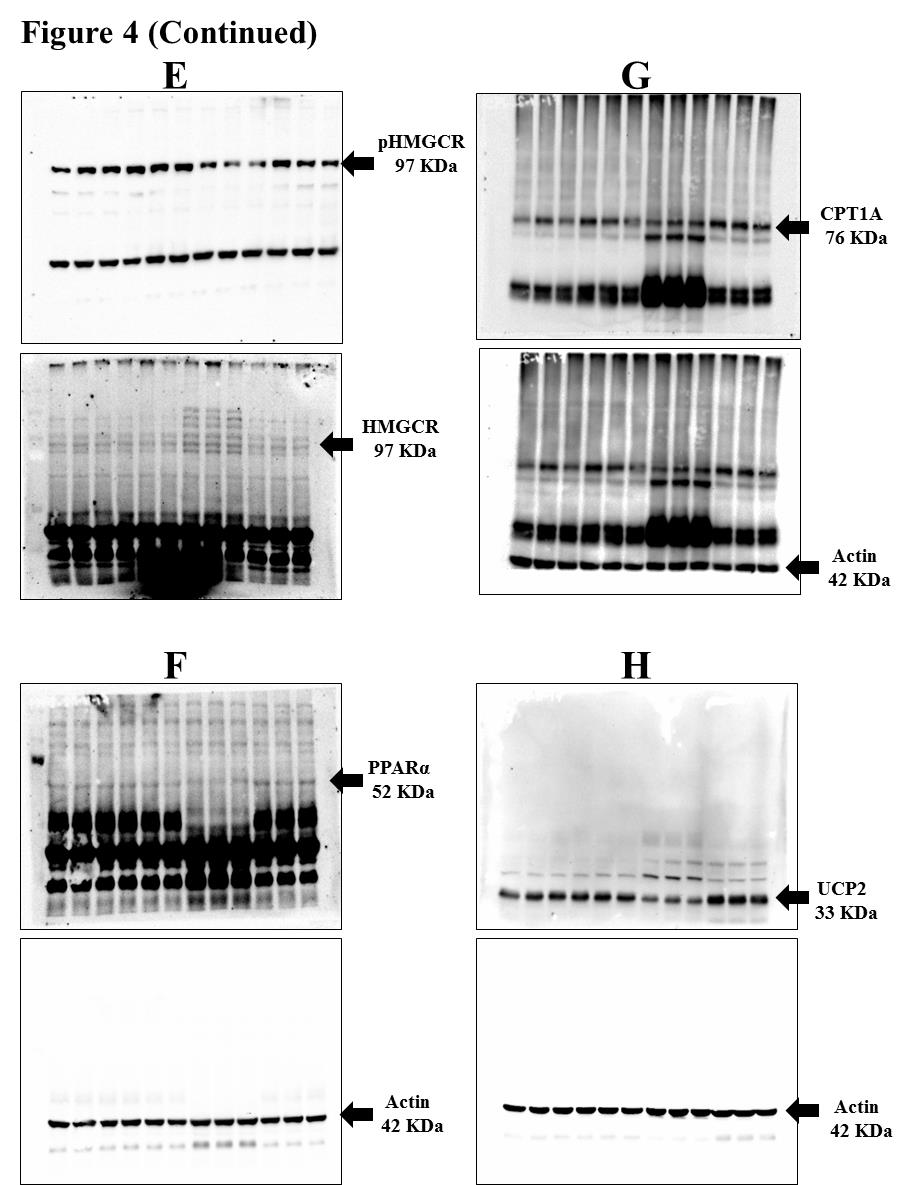

Supplement: Supplementary file 1 — Supplementary Dataset 1 [file 41598_2018_27607_MOESM1_ESM.docx]
